# SPG9A with the new occurrence of an *ALDH18A1* mutation in a CMT1A family with *PMP22* duplication: case report

**DOI:** 10.1186/s12883-021-02087-x

**Published:** 2021-02-11

**Authors:** Kishin Koh, Ryusuke Takaki, Hiroyuki Ishiura, Shoji Tsuji, Yoshihisa Takiyama

**Affiliations:** 1 Department of Neurology, Graduate School of Medical Sciences, University of Yamanashi, Yamanashi, 409-3898 Japan; 2 Department of Neurology, Iida Hospital, Nagano, 395-8505 Japan; 3 Department of Neurology, Graduate School of Medicine, The University of Tokyo, Tokyo, 113-8655 Japan; 4 Department of Molecular Neurology, University of Tokyo, Graduate School of Medicine, Tokyo, 113-8655 Japan; 5 Department of Neurology, International University of Health and Welfare, Chiba, 286-8686 Japan

**Keywords:** SPG9A, *ALDH18A1*, de novo mutation, Gonadal mosaicism, Charcot-Marie-tooth disease, *PMP22*

## Abstract

**Background:**

*ALDH18A1* mutations lead to delta-1-pyrroline-5-carboxylate-synthetase (P5CS) deficiency, which is a urea cycle-related disorder including SPG9A, SPG9B, autosomal dominant cutis laxa-3 (ADCL3), and autosomal recessive cutis laxa type 3A (ARCL3A). These diseases exhibit a broad clinical spectrum, which makes the diagnosis of P5CS deficiency difficult. We report here a rare Japanese family including both patients with an *ALDH18A1* mutation (SPG9A) and ones with CMT1A.

**Case presentation:**

A Japanese family included five patients with the CMT phenotype and five with the HSP phenotype in four generations. The patients with the HSP phenotype showed a pure or complicated form, and intrafamilial clinical variability was noted. Genetically, FISH analysis revealed that two CMT patients had a *PMP22* duplication (CMT1A). Exome analysis and Sanger sequencing revealed five HSP patients had an *ALDH18A1* heterozygous mutation of c.755G > A, which led to SPG9A. Haplotype analysis revealed that the *ALDH18A1* mutation must have newly occurred. To date, although de novo mutations of *ALDH18A1* have been described in ADCL3A, they were not mentioned in SPG9A in earlier reports. Thus, this is the first SPG9A family with a de novo mutation or the new occurrence of gonadal mosaicism of *ALDH18A1*. Analysis of serum amino acid levels revealed that two SPG9A patients and two unaffected family members had low citrulline levels and one had a low level of ornithine.

**Conclusions:**

Since the newly occurring *ALDH18A1* mutation, c.755G > A, is the same as that in two ADHSP families and one sporadic patient with SPG9A reported previously, this genomic site might easily undergo mutation. The patients with the c.755G > A mutation in our family showed clinical variability of symptoms like in the earlier reported two families and one sporadic patient with this mutation. Further studies are required to clarify the relationship between the amino acid levels and clinical manifestations, which will reveal how P5CS deficiency influences disease phenotypes including ARCL3A, ADCL3, SPG9B, and SPG9A.

## Background

SPG9A and SPG9B are hereditary spastic paraplegias (HSPs) caused by dominant and recessive mutations of *ALDH18A1*, respectively. These diseases are classified as delta-1-pyrroline-5-carboxylate-synthedase (P5CS) deficiency [1], which is a urea cycle-related disorder. P5CS deficiency includes SPG9A [OMIM #601162], SPG9B [OMIM #616586], ADCL3 [OMIM #616603], and ARCL3A [OMIM #219150] [2]. The disease severity in P5CS deficiency might increase in the order of SPG9A < SPG9B < ADCL3 ≤ ARCL3A [2]. These diseases can be understood with the concept of a disease continuum caused by various levels of loss of P5CS function [2]. P5CS deficiency exhibits a broad clinical spectrum including cutis laxa, connective tissue weakness, global developmental delay, microcephaly, cataracts, vomiting, hypotonia, spasticity, pyramidal signs, motor disability, and corpus callosum hypotrophy with variable disease severity. Thus, it is difficult to diagnose P5CS deficiency only from clinical information.

We describe here a rare Japanese family with various neurological manifestations including neuropathy, pure HSP, and complicated HSP. Although these phenotypes were initially considered as a consequence of a disease, molecular genetic analysis revealed there are two diseases, SPG9A and Charcot–Marie–Tooth disease type 1A (CMT1A), in one family. In Japan, although SPG9B has been reported in two families so far [3, 4], this is the first family with SPG9A. Furthermore, haplotype analysis indicated SPG9A might have been caused by the new occurrence of an *ALDH18A1* mutation in this family.

## Case presentation

### Patients and methods

**Fig. 1:**
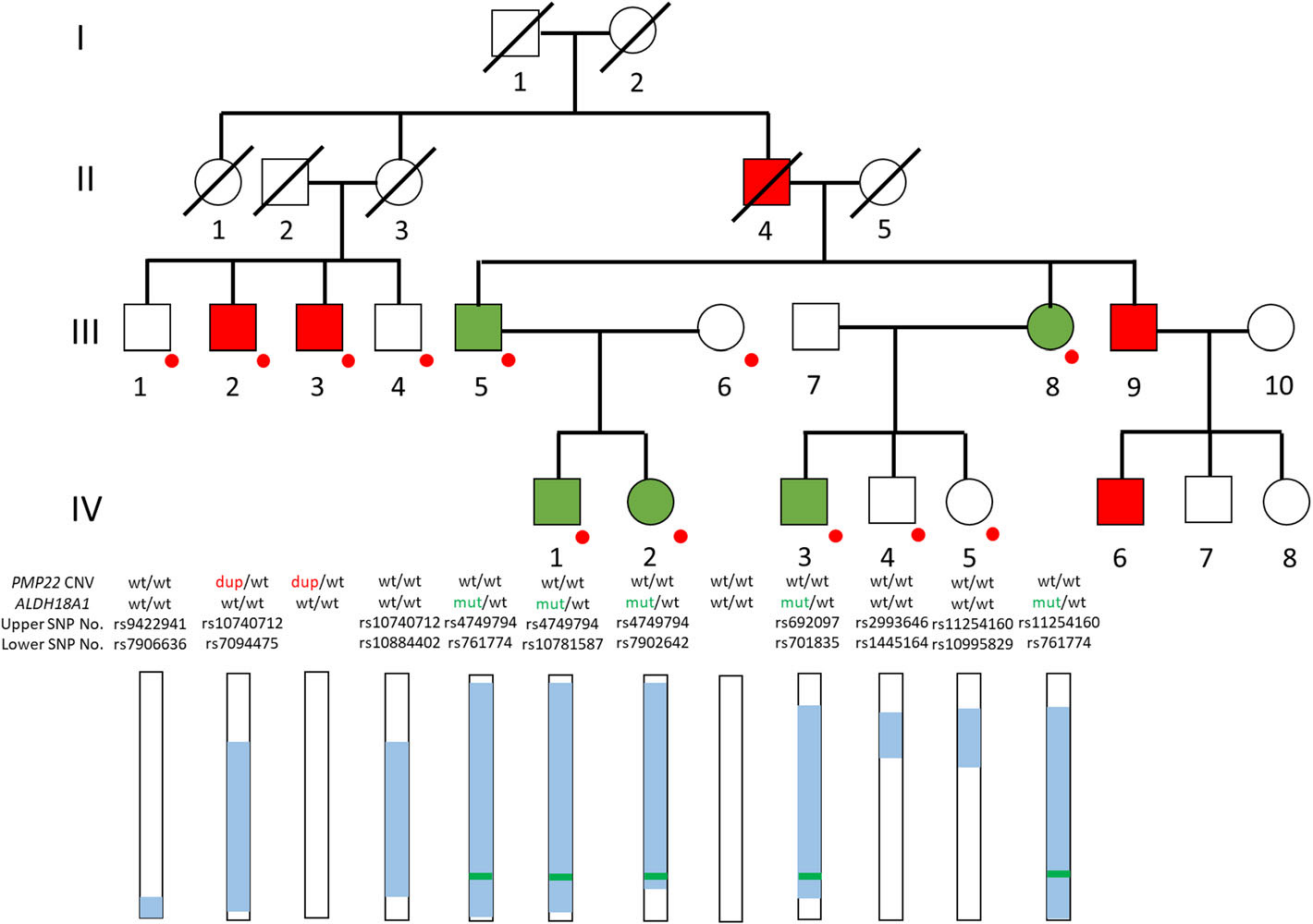
Squares and circles indicate males and females, respectively. Red and green filling indicates a CMT phenotype and HSP phenotype, respectively. Red dots indicate participants from which DNA was taken. Rectangles with white or/and light blue shading indicate the haplotypes of chr17, which were the inferred haplotypes. Light blue indicates the same haplotype. The upper and lower limits of the SNP for each blue haplotype are denoted by “Upper or Lower SNP No.” The green bars indicate the *ALDH18A1* mutation and its position. wt: wild type, dup: *PMP22* duplication, mut: *ALDH18A1* mutation

A pedigree chart is shown in Fig. 1. The clinical findings for each phenotype in representative patients are as follows.

Patient III-3 (68-year-old male) presented a phenotype of CMT. He showed gait disability when he was an elementary school student. In his 40s he exhibited clumsiness in his hands. These symptoms were slowly progressive. Neurological examination at age 68 revealed a steppage gait and his legs had the inverted champagne bottle appearance. The distal muscle power was moderately reduced. Tendon reflexes of the upper and lower limbs were decreased. Babinski signs were absent. A standardized electrophysiological study showed decreased conduction velocities of less than 38 m/s in the median and tibial nerves.

Patient IV-1 (38-year-old male) presented a phenotype of a pure form of HSP. He showed a spastic gait when he was a high school student. Neurological examination at age 38 revealed a spastic gait, increased deep tendon reflexes of the lower limbs, and positive Babinski signs.

Patient IV-2 (36-year-old female) presented a phenotype of a complicated form of HSP. She showed gait disability when she was an elementary school student. In her 30s she exhibited dysarthria and dysphagia. Neurological examination at age 36 revealed a spastic and ataxic gait, and positive Babinski signs. She did not show any muscle weakness.

In summary, Patients II-4, III-2, III-3, III-9, and IV-6 had a phenotype of CMT, Patients III-5, III-8, IV-1, and IV-3 a phenotype of a pure form of HSP, and Patient IV-2 a phenotype of a complicated form of HSP (Table 1).

**Table 1:**
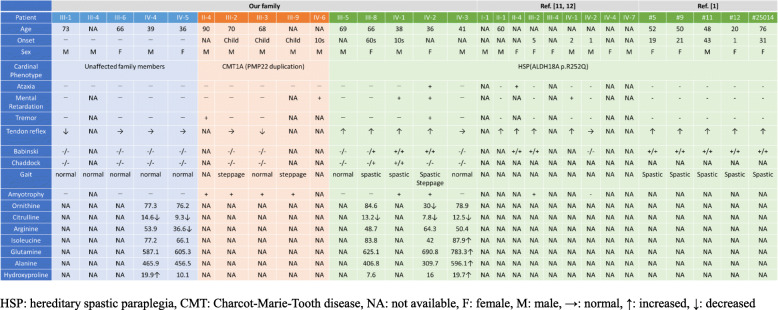
Clinical features and serum amino acid levels of the patients in the present family and those in the earlier reported families

*HSP* hereditary spastic paraplegia, *CMT* Charcot-Marie-Tooth disease, *NA* not available, *F* female, *M* male, →: normal, ↑: increased, ↓: decreased

We screened for *PMP22* duplications/deletions, which cause CMT1A, by fluorescence in situ hybridization (FISH) at first because we clinically diagnosed Patient III-3 as having a CMT1 phenotype. After screening for CMT1A, we performed target-sequencing for known HSP and CMT causative genes (Table 2) in Patients III-3 and IV-2. We filtered variations by using dbSNP146 [5], 1000 Genomes [6], HGVD [7], and iJGVD [8]. We evaluated the functional prediction of *ALDH18A1* mutations by means of in silico algorithms using the Combined Annotation Dependent Depletion (CADD) score [9]. We applied Sanger sequencing to perform a co-segregation study in this family. Furthermore, we performed haplotype analysis by using SNP typing with Affymetrix Genome-Wide Human SNP array 6.0. Haplotypes were reconstructed with Allegro v2 [10]. In addition, we determined the serum amino acid levels in Patients III-8, IV-2, 3, 4, and 5.

**Table 2:** HSP and CMT genes checked

(*ATL1, SPAST, NIPA1, KIAA0196, ALDH18A1, KIF5A, RTN2, HSPD1, BSCL2, ATSV, REEP1, ZFYVE27, SLC33A1, REEP2, CPT1C, CYP7B1, SPG7, SPG11, ZFYVE26, ERLIN2, SPG20, ACP33, B4GALNT1, DDHD1, FA2H, PNPLA6, c9orf12, GJA2, AP4B1, KIAA0415, TECPR2, AP4M1, AP4E1, AP4S1, VPS37A, DDHD2, c12orf65, CYP2U1, TFG, KIF1C, USP8, WDR48, ARL6IP1, ERLIN1, AMPD2, ENTPD1, ARSI, PGAP1, FLRT1, RAB3GAP2, MARS, ZFR, IBA57, MAG, MPZ, LITAF, EGR2, NEFL, FBLN5, KARS, SOX10, GJB3, ARHGEF10,G GNB4, HARS, GDAP, MTMR2, SBF2, SBF1, SH3TC2, NDRG1, EGR2, PRX, HK1, FGD4, FIG 4, SURF1, CTDP1, ASAH1, PMM2, GALC, ARSA, PHYH, PEX7, ABHD12, DNAJC3, GJB1, MFN2, KIF1B, RAB7, TRPV4, GARS, HSPB1, GDAP1, HSPB8, DNM2, AARS, DYNC1H1, LRSAM1, DHT, DNAJB2, MARS, NAGLU, HARS, VCP, MORC2, LMNA, MED25, HSPB1, DNM2, YARS, INF2, GNB4, GDAP*)	

## Results

We identified duplication of *PMP22* in Patients III-2 and III-3, who presented a CMT phenotype on FISH analysis. On whole exome analysis in Patient IV-2, we identified two candidate variations in *GARS* (c.374A > T, p.E125V) and *ALDH18A1* (c.755G > A, p.R252Q) in Patient IV-2. The variation in *GARS* did not co-segregate with CMT patients in this family. On the other hand, the *ALDH18A1* variation co-segregated with patients with a phenotype of HSP (Patients III-5, 8, IV-1, IV-2, and IV-3). This variation was not detected in dbSNP146 [5], 1000 genome project [6], HGVD [7], or iJGVD [8], and the CADD score was 26.8 (deleterious > 20). Moreover, this variation was previously reported as a disease-causing variant in SPG9A [1, 11–13]. These results showed Patients III-2 and III-3 had CMT1A, and Patients III-5, III-8, IV-1, IV-2, and IV-3 had SPG9A. Furthermore, haplotype analysis revealed that individuals I-1, II-3, II-4, III-2, III-4, III-5, III-8, and IV-1-3 had the same haplotype including the *ALDH18A1* mutation position (Fig. 1). However, the *ALDH18A1* mutation was only identified in individuals III-5, III-8, IV-1, IV-2, and IV-3. This indicated that the *ALDH18A1* mutation may have occurred as a de novo mutation on transmission to individual II-4 from either the parents or the new occurrence of gonadal mosaicism.

Analysis of serum amino acid levels revealed that all participants including three SPG9A patients and two unaffected family members had low levels of citrulline and Patient IV-2 had low levels of citrulline and ornithine.

## Discussion and conclusions

This study revealed SPG9A patients and CMT1A ones in one family. Haplotype analysis revealed that an *ALDH18A1* mutation might have newly occurred as a de novo mutation from the 1st generation to Patient II-4 or the new occurrence of gonadal mosaicism in Patient II-4. To date, although de novo mutations of *ALDH18A1* have been described in ADCL3A [14–16], they were not reported in SPG9A in earlier reports [1, 11, 17]. Thus, this is the first SPG9A family with a de novo mutation or the new occurrence of gonadal mosaicism of *ALDH18A1*. It was transmitted to five individuals with a HSP phenotype.

Since the newly occurring mutation of *ALDH18A1*, c.755G > A, has been reported in two families and one sporadic case so far [1, 11, 13], this genetic site might be important because it was shown that the c.755G > A mutation promotes the dissociation of a hexamer into dimers [11]. This increased tendency of the c.755G > A mutant form of P5CS to dissociate must reflect disturbance by the mutation of the intersubunit interactions within the hexamer. According to in silico analysis and the previous report, this mutation is disease-causing. SPG9A was reported to have both pure and complicated forms [1, 4]. It was also reported that the same *ALDH18A1* mutation in the same family caused variability of symptoms [1]. The present family also had pure and complicated forms of HSP. The previously reported two families and one sporadic case also exhibited variability of symptoms, i.e., pure or complicated forms, severe or mild. Therefore, it seems to be difficult to determine the genotype-phenotype correlation in SPG9A. However, the *ALDH18A1* mutation would exhibit a genotype-phenotype correlation because *ALDH18A1* deficiency shows several phenotypes, i.e., SPG9A, SPG9B, ADCL3, and ARCL3, which reflect the P5CS function. Fluctuation of the serum amino acid levels was reported in ADCL3 [16]. It might be considered that fluctuation of the serum amino acid levels would reflect severity and mutation would reflect P5CS deficiency.

Our data show that all participants with the *ALDH18A1* mutation had low citrulline levels, although individuals without the mutation also showed low citrulline levels. On the other hand, only one patient with a low ornithine level had a complicated form of HSP. However, one report on *ALDH18A1* mutations showed instability of the serum amino acid levels [7]. Therefore, further studies are required to clarify the relationship between the amino acid levels and clinical manifestations. This will reveal how P5CS deficiency influences disease phenotypes including ARCL3A, ADCL, SPG9B, and SPG9A.

## Data Availability

The data used and/or analyzed during the current study are available from the corresponding author on reasonable request.

## Abbreviations

HSP: Hereditary spastic paraplegia
P5CS: Delta-1-pyrroline-5-carboxylate-synthetase
CMT1A: Charcot–Marie–Tooth disease type 1A
FISH: Fluorescence in situ hybridization
